# SNP-SNP Interactions Discovered by Logic Regression Explain Crohn's Disease Genetics

**DOI:** 10.1371/journal.pone.0043035

**Published:** 2012-10-12

**Authors:** Irina Dinu, Surakameth Mahasirimongkol, Qi Liu, Hideki Yanai, Noha Sharaf Eldin, Erin Kreiter, Xuan Wu, Shahab Jabbari, Katsushi Tokunaga, Yutaka Yasui

**Affiliations:** 1 School of Public Health, University of Alberta, Edmonton, Alberta, Canada; 2 Department of Human Genetics, School of International Health, Graduate School of Medicine, University of Tokyo, Tokyo, Japan; 3 Fukujuji Hospital, Japan Anti-Tuberculosis Association, Kiyose, Japan; Dr. Margarete Fischer-Bosch and University of Tübingen, Germany

## Abstract

In genome-wide association studies (GWAS), the association between each single nucleotide polymorphism (SNP) and a phenotype is assessed statistically. To further explore genetic associations in GWAS, we considered two specific forms of biologically plausible SNP-SNP interactions, ‘SNP intersection’ and ‘SNP union,’ and analyzed the Crohn's Disease (CD) GWAS data of the Wellcome Trust Case Control Consortium for these interactions using a limited form of logic regression. We found strong evidence of CD-association for 195 genes, identifying novel susceptibility genes (e.g., *ISX*, *SLCO6A1*, *TMEM183A*) as well as confirming many previously identified susceptibility genes in CD GWAS (e.g., *IL23R*, *NOD2*, *CYLD*, *NKX2-3*, *IL12RB2*, *ATG16L1*). Notably, 37 of the 59 chromosomal locations indicated for CD-association by a meta-analysis of CD GWAS, involving over 22,000 cases and 29,000 controls, were represented in the 195 genes, as well as some chromosomal locations previously indicated only in linkage studies, but not in GWAS. We repeated the analysis with two smaller GWASs from the Database of Genotype and Phenotype (dbGaP): in spite of differences of populations and study power across the three datasets, we observed some consistencies across the three datasets. Notable examples included *TMEM183A* and *SLCO6A1* which exhibited strong evidence consistently in our WTCCC and both of the dbGaP SNP-SNP interaction analyses. Examining these specific forms of SNP interactions could identify additional genetic associations from GWAS. R codes, data examples, and a ReadMe file are available for download from our website: http://www.ualberta.ca/~yyasui/homepage.html.

## Introduction

Analysis of genome-wide association studies (GWAS) often focuses on identifying individual single nucleotide polymorphisms (SNPs) that modify the risk of a phenotype, assuming the underlying association of an individual SNP without considering the involvement of any other SNPs. GWASs of Crohn's Disease (CD) have also focused largely on finding such *marginal* associations of individual SNPs, where the association of each SNP with CD risk is evaluated without considering other SNPs. If individual SNPs (or the regions tagged by them) are independently critical in the CD-risk-altering biological functions, this approach would be effective. This may be the case for the association between rs11209026 of the *IL23R* gene and CD risk, where the marginal association is quantified as an estimated odds ratio of 2.66^1^. The other SNPs that are statistically significantly associated with CD risk, however, show very weak associations with estimated odds ratios typically in the range of less than 1.5. In addition, the sum of such marginal associations is far from describing the estimated degree of genetic contributions to the risk of CD [1]. A possible explanation for this may be that an individual SNP (or a region tagged by it) is not independently critical in the biological functions that affect the CD-risk: rather, interaction among multiple SNPs (or regions tagged by them) may *jointly* affect the CD-risk. Specifically, the following two forms of SNP-SNP interactions may be motivated biologically. One is the *SNP intersection* form using the set theory terminology, in which *all of* the SNPs in a set must take their respective high-risk genotypes for CD risk to be elevated, where one, or a subset, of the set is insufficient to influence CD-risk-altering biological functions. That is, an increase in the CD-risk requires all SNPs (i.e., SNP-A *and* SNP-B *and* …) to take their respective high-risk genotypes. This form of interaction is similar to a set of sequential mutations that must accumulate before a cell transforms in the multistage carcinogenesis theory. The other form of SNP-SNP interaction that is biologically plausible is motivated by the notion of genetic heterogeneity. Specifically, CD risk may be elevated through multiple independent ways, each of which may be a SNP-intersection or an individual SNP (i.e., (SNP-A *and* SNP-B) *or* (SNP-C) each taking its respective high-risk genotype. This form of SNP-SNP interaction is referred to as a *SNP union*, also derived from the set theory terminology. Under a single or combination of SNP intersections and SNP unions, assessing the independent *marginal* effect of each individual SNP without considering these interaction forms will either fail to discover, or observe only weak, association between the individual SNP and the phenotype of interest.

To incorporate these specific forms of SNP-SNP interactions in GWAS data analysis, we propose using logic regression to search for sets of SNPs that are jointly associated with the phenotype of interest in the form of a single SNP intersection or union, or in combinations of thereof [2]. Logic regression is an innovative and powerful statistical learning technique that is used to model an outcome (e.g., the disease status in a case-control study) with intersections and/or unions of multiple potential predictors that are primarily binary, such as indicators of SNP genotypes (i.e., indicators of the minor-allele homozygous and indicators of the heterozygous and the minor-allele homozygous). As such, logic regression can select a model that may involve various intersections and/or unions of SNPs within a gene, or any set of SNPs (e.g., SNPs of genes in a certain biological pathway), that are associated with a phenotype. Logic regression has been applied successfully to a number of SNP data analyses *with selected candidate genes* [3]–[11]. To our knowledge, however, it has not been applied to GWAS analysis due to the considerable computational demands in searching for SNP intersection/union combinations among a large number of SNPs in GWASs.

## Materials and Methods

### Incorporating specific forms of SNP-SNP interactions in GWAS

Our logic-regression-based gene-level SNP-SNP-interaction analysis of GWAS data can be summarized as follows. Combinations of SNP intersections and unions can be expressed mathematically as Boolean combinations, such as (X1 ∧ X2) ∨ X3^c^, where “∧”, “∨”, and “^c^” represents intersection (AND), union (OR), and complement (NOT), and X's are indicators of SNP genotypes. The logic regression model takes the form:
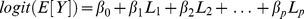
where Y is a binary phenotype, CD cases versus controls, β_0_, β_1_,… β_p_ are the parameters, and L_1_, L_2,_ …, L_p_ are Boolean combinations of genotype indicators of SNPs within a gene, also called logic trees. The logic trees are selected adaptively, using a Simulated Annealing algorithm, and based on deviance as the model fit measure [2]. Our logic-regression-based SNP-SNP interaction analysis uses genes with at least two genotyped SNPs. To reduce redundancy of logic trees that genotype indicators of SNPs within a gene can form, we removed SNPs within each gene sequentially, before logic regression, such that no pair of remaining SNPs within a gene had linkage disequilibrium (r^2^≥0.8). In each logic regression fit, we allowed a maximum of two Boolean combinations (Ls) of at most five indicators of SNP genotypes in total. Note that these constraints are necessary in GWAS because logic regression must search a large number of potential combinations, and therefore comes with a high computational cost. To correct for the inherent instability of the performance measure when searching a large space, we refit the logic regression 20 times, starting the algorithm with 20 different initial values: this process was applied to the original dataset as well as 20 datasets obtained by permutations of the case- control labels. Of the 20 results produced by the 20 starting values, we selected the best fit, measured by deviance.

### Measure of evidence of association

Running logic regression for each gene in the original dataset, as well as their 20 case-control-label permuted datasets, yields an approximate Bayes Factor (BF) for each gene. The BF is approximated by the corresponding Likelihood Ratio in this case (which eliminates the need to specify priors, similar to the approximation used by Bayesian Information Criterion for BF), in the base-10 logarithm (equivalent to LOD Score), where the denominator is the median of 19 (log10) maximum likelihoods from the 19 permuted datasets (20 minus one because BF of a permuted dataset should not use its own BF in calculating the median of BF from the permuted datasets). An important feature of this approximate BF is that the denominator standardizes for the higher potential for genes with larger numbers of SNPs to overfit. We follow the Wellcome Trust Case Control Consortium (WTCCC) 's framework of using BF as the measure of evidence of the observed association between each gene and CD risk^12^. Specifically, suppose we have N genes to be investigated, of which 10 genes are assumed to be truly associated with CD risk. The prior odds for CD-risk association for any gene is therefore 10/(N-10). To make the posterior odds of CD-risk association for a gene to 10 (i.e., probability that the gene is associated with CD risk is 10/11, or approximately 0.91), a likelihood ratio for the association over no association (i.e., the BF under the same-size logic-regression model) has to be (N-10). Based on the number of genes we examined in the WTCCC dataset (13,106 after mapping), the WTCCC framework above specifies a BF of 4.12 as the threshold, above which there is strong evidence of association between the gene and CD risk. The P-value for each gene is calculated as the proportion of all permuted BF values of *all genes* larger than the gene's observed BF. This p-value calculation properly takes the multiple testing into account.

We checked if our BF-based hypothesis testing has a proper size (i.e. control over the false positive rate) by using a simulation study. We randomly chose 200 genes from Chromosome 1 (Chromosome 1 contains approximately 1,300 genes after mapping). We simulated a total of 50 null hypothesis datasets by shuffling case-control labels randomly and imposing an equal number of cases and controls in each dataset. We ran the logic-regression-based SNP-SNP interaction analysis and estimated p-value for each of the 200 genes in each of the 50 null datasets. The 10,000 p-values roughly followed a uniform distribution (data not shown), indicating that our testing procedure has a proper size and proper control over the false positive rate.

### WTCCC and dbGaP Studies

We applied the logic-regression-based SNP-SNP interaction analysis method to the WTCCC's GWAS data comparing 2,005 CD cases to the 1,502 members of the British 1958 birth control cohort (58C) plus the 1,500 controls of the UK Blood Service sample: these used Affymetrix GeneChip Human Mapping 500K Array Sets [12]. We also repeated the analysis with two much smaller GWASs: the Database of Genotype and Phenotype (dbGaP) non-Jewish case-control GWAS data on 513 cases and 515 controls; and dbGaP Jewish case-control GWAS data on 300 cases and 432 controls. The dbGaP GWAS used the Illumina Sentrix HumanHap300 Genotyping BeadChip [13]. For exclusion of cases and controls from the WTCCC analysis, we followed the WTCCC's recommendations based on the sample call rates and evidence of recent non-European ancestry [12]. Specifically, 24 control subjects were excluded from the 1958 British birth cohort of controls, 42 control subjects were excluded from the UK Blood Service cohort of controls, and 257 CD cases were not used in the analysis. In the two dbGaP analyses, we removed subjects with sample call rates less than 95% (15 controls and 17 cases were excluded from the non-Jewish data, and three controls and nine cases were excluded from the Jewish data).

The genotype calls of the WTCCC were generated by the Chiamo calling algorithm. Following the WTCCC's recommendations, we only considered genotype calls with confidence score >0.9, and treated the rest of the calls as missing genotypes. SNPs with SNP call rates less than 95% were removed. We also removed SNPs based on their minor allele frequencies: the default minor allele frequency cutoff in the GenABEL R package was used (2.5/N where N is the number of subjects), resulting in cutoffs of 0.05% for the WTCCC database and 0.3% for the Jewish and non-Jewish dbGaP databases. We used a cutoff of 0.2 for the Hardy-Weinberg Equilibrium (HWE) test's false discovery rates, based on controls. SNP-gene mapping files were retrieved from the OpenBioinformatics website (http://www.openbioinformatics.org/gengen/tutorial_calculate_gsea.html#_Toc210887414).

We checked the homogeneity of the three populations, WTCCC, dbGaP non-Jewish and Jewish, by running Principal Component Analysis using the R package GenABEL [14]. We computed a matrix of genomic kinship between all pairs of subjects, based on the 22,498 SNPs common to the three datasets. More specifically, we calculated the average Identity-by-State (IBS) for the 6,400 subjects (4,684, 720, and 996 subjects from the WTCCC, dbGaP non-Jewish, and dbGaP Jewish studies, respectively), as the pairwise similarity measure. We then performed Principal Component Analysis based on the pairwise-similarity matrix of average IBS. A plot of the first two principal components, displayed in Figure S1, suggests that dbGaP Jewish population was genetically quite different from the WTCCC and the dbGaP non-Jewish populations, but the latter two populations also showed some appreciable between-population differences and within-population heterogeneity (Figure S1).

## Results

There were 195 genes with strong evidence of association between the gene and CD risk in the logic-regression gene-level SNP-SNP-interaction analysis of the WTCCC GWAS data, 40 of which are listed in Table 1 (all are shown in Table S1). Notably, all nine regions of the genome showing strong evidence of association by the single-SNP analysis of WTCCC data^12^, as well as seven out of the eight regions showing moderate evidence of association, were represented among the 195 genes. Thirty-seven (63%) of the 59 chromosomal locations, that were previously identified by a meta-analysis of single-SNP studies that involved over 22,000 cases and 29,000 controls [1], were included in the 195 genes. Also included in the 195 genes that showed strong evidence of association were three genes located in *IBD1* (Chr 16q12), two genes in *IBD2* (Chr 12q13), six genes in *IBD3* (Chr 6p21, HLA region), eight genes in *IBD5* (Chr 5q31-33), two genes in *IBD6* (Chr 19p13), and one gene in *IBD7* (Chr 1p36), well-established regions of chromosomes for CD risk: no gene in *IBD4* (Chr 14q11-12) was included, however. In addition, there were a number of chromosome regions that did not show strong or moderate evidence of association in the single-SNP analysis of WTCCC, but had three or more genes appearing among the 195 genes, namely, 1q32, 2q14, 8p12, 10q22, 10q26, 11p14, and 18q22. These are indicated by green highlighting in the tables. Furthermore, there are clusters corresponding to certain families of genes in the 195 genes. For example, genes associated with phosphoprotein phosphatase activity (e.g., *PPM1K*, *PPM1L*, *PPP2R2C*, *PTPN2*) showed strong evidence of association with CD risk, of which only *PTPN2* had been previously indicated.

**Table 1:** Forty genes with the strongest evidence for association with Crohn's Disease risk, with chromosomal locations, numbers of SNPs, approximate p-values, and Bayes factors.

Gene Name	Chromosome	#SNPs	p-value	C.BF
ISX	22q12	84	<3.8×10^−6^	148.5
SEMA6A*	5q23	152	<3.8×10^−6^	96.2
GTF3C4	9q34	4	<3.8×10^−6^	91.8
PTGFRN	1p13	15	<3.8×10^−6^	85.5
ADRA1B**	5q33	45	<3.8×10^−6^	82.3
MYLK3	16q11	2	<3.8×10^−6^	77.0
HTR3B	11q23	10	<3.8×10^−6^	75.7
RRP15	1q41	29	<3.8×10^−6^	75.4
RGL1	1q25	20	<3.8×10^−6^	69.9
SORBS1	10q23	46	<3.8×10^−6^	65.5
CALCOCO1	12q13	15	<3.8×10^−6^	57.9
TMEM156	4p14	13	<3.8×10^−6^	52.7
XRCC6BP1	12q14	38	<3.8×10^−6^	45.9
FXR1	3q28	7	<3.8×10^−6^	37.7
GARNL1	14q13	4	<3.8×10^−6^	34.9
GPR161*	1q24	7	<3.8×10^−6^	30.9
SORCS1**	10q23-q25	265	<3.8×10^−6^	30.6
SAC*	1q24	13	<3.8×10^−6^	28.4
LRP1B	2q21	241	<3.8×10^−6^	27.2
C18orf62	18q23	79	<3.8×10^−6^	25.9
CSRP1	1q32+	17	<3.8×10^−6^	24.2
POU6F2	7p14	58	<3.8×10^−6^	22.6
LEF1	4q23-q25	31	<3.8×10^−6^	22.3
SEL1L	14q31	170	<3.8×10^−6^	21.9
SVIP	11p14+	88	<3.8×10^−6^	21.7
VRK1	14q32	128	<3.8×10^−6^	19.3
GLRX3	10q26+	79	<3.8×10^−6^	18.4
ID4*	6p22	79	<3.8×10^−6^	15.3
CDH10	5p14	107	<3.8×10^−6^	14.9
NOD2**	16q21	5	<3.8×10^−6^	14.6
NHLRC1*	6p22	7	<3.8×10^−6^	14.0
FMN2	1q43	60	<3.8×10^−6^	14.0
IL23R**	1p31	11	<3.8×10^−6^	13.6
PTGER4**	5p13	46	<3.8×10^−6^	13.5
CTNNA3	10q22+	257	<3.8×10^−6^	13.3
PNPLA6	19p13	5	<3.8×10^−6^	13.0
FBXO15	18q22+	94	<3.8×10^−6^	12.5
ATG16L1**	2q37	7	<3.8×10^−6^	12.4
RTP2	3q27	4	<3.8×10^−6^	12.0
KCNIP4	4p15	154	<3.8×10-6	11.8

** indicates genes in the chromosomal locations where the WTCCC single-SNP analysis showed **strong** evidence.

* indicates genes in the chromosomal locations where the WTCCC single-SNP analysis showed **moderate** evidence.

+ indicates chromosomal locations are those with three or more genes in the 195 genes (see Table S1) showing strong evidence in our WTCCC logic-regression-based analysis, but without strong or moderate evidence in the single-SNP analysis of WTCCC.

Intestine Specific Homeobox (*ISX*) was the gene most strongly associated with CD risk in our WTCCC logic-regression-based analysis and represents a new CD susceptibility gene. Homeobox genes encode DNA-binding proteins, of which many are thought to be involved in early embryonic development. *ISX* is a transcription factor that regulates gene expression in the intestine [15]. The logic structure of *ISX* is shown in Table 2. Based on the genotypes of the SNPs in the two trees, the following three risk groups shown in Table 2 emerge: a reference risk group (1540 cases/2562 controls); a low risk group (1 cases/372 controls, estimated odds ratio 0.0045); and a high risk group (207 cases/2 controls, estimated odds ratio 172.2). Both the low and high risk groups are defined by uncommon variants with over 150-fold effect sizes. We confirmed the allele frequencies of these SNPs with the Hapmap CEU population as an informal check of the possibility of genotyping errors for the rare variants.

**Table 2:** Logic structures, frequencies, and associated Crohn's Disease odds ratios of the *ISX* gene (p-value<3.8×10^−6^).

		rs11089728CC	rs9610191 TT	rs17778240 TT	rs17778240 TT	rs5999715AC	Logic-based Risk Groups
Genotype Freq	Case N = 1748	797 (45.6%)	10 (0.6%)	466 (26.7%)	466 (26.7%)	214 (12.2%)	
	Cont N = 2936	1326 (45.2%)	18 (0.6%)	776 (26.4%)	776 (26.4%)	17 (0.6%)	

Logic 1	AND (OR)		Frequency		Odds Ratio
Logic 2		AND	Case	Cont	
Logic-based Risk Groups	Logic 1 = No	Logic 2 = No	1540	2562	1.0
	Logic 1 = Yes	Logic 2 = No	1	372	.0045
	Logic 1 = No	Logic 2 = Yes	2	0	172.2
	Logic 1 = Yes	Logic 2 = Yes	205	2	

We note that using the WTCCC dataset for discovery and the dbGaP non-Jewish and Jewish datasets for replication is untenable, because of the observed population differences (Figure S1) and the difference in study power due to the large differences in sample sizes. Another disadvantage is the difference in genotyping platforms between these data sets, including their genotyping errors and genomic coverage. Nonetheless, we applied the same method of analysis to the dbGaP's non-Jewish and Jewish GWAS datasets. Since this analysis focused on the 195 genes with strong evidence of association with CD risk in the WTCCC analysis, the BF threshold for strong evidence for this stage of the analysis is 2.29. We applied this threshold to the larger BF of the two dbGaP GWAS analyses. Table 3 lists 17 genes that showed strong evidence for their CD-risk association in both stages of the analysis. Seven of the seventeen genes in Table 3 are located in regions of the genome that showed strong or moderate evidence of association with CD by the single-SNP analysis of WTCCC data [12]. Of the remaining ten genes, *TMEM183A* and *NEK2* are both located in Chromosome 1q32. Chromosome 1q32 has been shown to be associated with the risk of Ankylosing Spondylitis that is linked to CD [16]: this region was not identified by the single-SNP WTCCC or dbGaP analyses. A gene of organic anion transporter, *SLCO6A1*, showed strong evidence of association in all three GWASs, in spite of no previous implication of CD-risk association: this is significant in view of the known association of *SLC22A4* and *SLC22A5* (*IBD5*), genes of organic cation transporters, with CD risk [17]. In addition to *SLCO6A1*, three genes (*IL23R*, *NOD2*, and *TMEM183A*) showed consistently strong evidence across the three datasets.

**Table 3:** Seventeen genes with the strong evidence for association with Crohn's Disease risk in WTCCC and one or both of Non-Jewish and Jewish dbGap GWASs, with chromosomal locations, numbers of SNPs, approximate p-values, and Bayes factors.

GWAS Study Name		WTCCC			Non-Jewish dbGap			Jewish dbGap		
Sample Size (Cases/Controls)		(1748/2936)			(498/498)			(291/429)		
Gene Name	Chromosome	#SNPs	p-value	C.BF	#SNPs	p-value	C.BF	#SNPs	p-value	C.BF
IL23R**	1p31	11	<3.8×10^−6^	13.6	14	<3.8×10^−6^	9.3	18	1.4×10^−3^	3.8
NOD2**	16q21	5	<3.8×10^−6^	14.6	4	2.9×10^−5^	5.8	4	8.7×10^−3^	2.8
TMEM183A	1q32+	10	7.6×10^−5^	5.3	5	6.0×10^−4^	4.3	5	1.9×10^−3^	3.7
SLCO6A1	5q21	15	5.7×10^−5^	5.4	11	4.7×10^−4^	4.4	11	1.6×10^−2^	2.4
PTGER4**	5p13	46	<3.8×10^−6^	13.5	50	1.6×10^−4^	4.9	53	1.6×10^−1^	0.9
CYLD**	16q12	30	<3.8×10^−6^	11.2	22	2.2×10^−3^	3.6	21	1.1×10^−1^	1.2
SOCS6	18q22+	111	3.4×10^−5^	5.6	147	4.7×10^−2^	1.8	145	1.5×10^−2^	2.5
ACAD11	3q22	4	<3.8×10^−6^	6.2	5	3.7×10^−1^	0.3	5	1.2×10^−3^	3.9
CLSTN2	3q23	120	<3.8×10^−6^	6.3	100	4.0×10^−4^	4.4	104	7.6×10^−1^	−0.5
SOX11	2p25	194	<3.8×10^−6^	9.6	194	2.8×10^−3^	3.4	188	3.0×10^−1^	0.4
CEBPB	20q13	15	5.9×10^−4^	4.2	27	2.2×10^−1^	0.7	28	5.5×10^−3^	3.1
C1orf141**	1p31	10	<3.8×10^−6^	10.3	6	4.1×10^−3^	3.2	7	3.5×10^−1^	0.3
NEK2	1q32-q41+	11	8.8×10^−5^	5.1	13	4.0×10^−1^	0.2	12	4.9×10^−3^	3.1
NKX2-3**	10q24	14	7.6×10^−6^	6.0	7	8.4×10^−3^	2.8	7	2.9×10^−1^	0.5
BSN**	3p21	4	<3.8×10^−6^	7.0	3	1.2×10^−2^	2.6	3	7.0×10^−1^	−0.4
RBMS3	3p24-p23	157	2.1×10^−4^	4.7	177	8.8×10^−1^	−0.8	175	6.9×10^−3^	2.9
C10orf57	10q22+	6	6.3×10^−4^	4.2	9	8.1×10^−1^	−0.6	9	1.4×10^−2^	2.5

** indicates genes in the chromosomal locations where the WTCCC single-SNP analysis showed **strong** evidence.

+ indicates chromosomal locations are those with three or more genes in the 195 genes (see Table S1) showing strong evidence in our WTCCC logic-regression-based analysis, but without strong or moderate evidence in the single-SNP analysis of WTCCC.

## Discussion

Our results illustrate the power of the logic-regression-based GWAS analysis in identifying specific forms of SNP-SNP interactions associated with a phenotype and explaining a greater extent of CD genetics. We found strong evidence of CD-Association with 195 genes including both previously identified loci through the single-SNP analysis, in addition to newly identified susceptibility genes.

In this paper, we reduced the computational demand of logic regression by limiting the search to SNP combinations within the same gene, and also by fixing the size of SNP combinations in the search. These strategies have a definite disadvantage: the search will not be comprehensive and true underlying SNP-SNP interactions that are more complex than the limited size under consideration will not be discovered. In view of the current practice of assessing the marginal effects of individual SNPs one at a time, however, we submit that the limited form of logic regression proposed here provides a clear advance over, and an alternative to, the individual-SNP analysis. It can search for more biologically-plausible forms of SNP effects (combination of SNP intersections and/or unions) with greater degrees of association indicated by appreciably larger values of odds ratios, although the search remains approximate due to the limited size.

Despite the limitation of our approach by the small fixed size of logic regression models, the successful discovery of CD susceptibility genes demonstrates the potential utility of the logic-regression-based SNP-SNP interaction analysis of GWAS in providing additional insights to the *marginal* single-SNP analysis approach of GWAS. Some of the genes (or their chromosomal regions) identified by our approach were previously identified only in linkage studies and not by GWAS: this also attests to the utility of the proposed approach.

False positive discoveries by GWAS in which a large number of SNPs are examined for association with a disease are a major concern. Any discoveries including those reported here have to be validated rigorously in further investigations for exclusion of false positive from population stratification and genotyping errors. The candidate gene approach is also a valid alternative to the data-driven approach of GWAS, whether driven by a functional or biological hypothesis or possibly following the potential discoveries of GWAS. The application of logic regression is less computationally involved in candidate-gene studies, compared to GWAS.

Proper phenotyping is a key for increasing the chance to identify susceptibility genes specific for a clinical phenotype of interest. A recent paper on Crohn's disease [18] provided a good example on this point: it focused specifically on the small intestinal inflammation phenotype of the disease and showed that impairments in Wnt signalling and Paneth cell biology are pathophysiological hallmarks of this clinical phenotype.

Increasing attention has been paid recently to pathway-based analysis of GWAS [19]. Our approach can be extended from gene-level SNP-SNP interaction to pathway-level SNP-SNP interaction. This approach would be biologically appealing, as it uses sets of SNPs within the same pathway rather than within the same gene; however, the logic space to be explored in the pathway-level analysis is appreciably larger than in the gene-level counterpart. The search space may be restricted based on some biological criteria, such as restricting the search to non-synonymous coding variants. Such SNP-SNP interaction analysis at the pathway level could, however, provide further valuable insights into genetic interactions on the modification of phenotype risks.

## Supporting Information

Table S1 **One hundred and ninety five genes with the strongest evidence for association with Crohn's Disease risk, with chromosomal locations, numbers of SNPs, approximate p-values, and Bayes factors.**
(DOC)

Figure S1 **Principal components from the three datasets: WTCCC, Non-Jewish and Jewish subjects are represented by black circles, green pluses, and blue triangles, respectively.**
(PDF)
