# Supplementary material for: SNP-SNP Interactions Discovered by Logic Regression Explain Crohn's Disease Genetics
Source: PLoS One. 2012 Oct 12;7(10):e43035. doi: 10.1371/journal.pone.0043035 (PMC3470545; doi:10.1371/journal.pone.0043035)
Supplement: Table S1 — One hundred and ninety five genes with the strongest evidence for association with Crohn's Disease risk, with chromosomal locations, numbers of SNPs, approximate p-values, and Bayes factors. (DOC) [file pone.0043035.s001.doc]

**Table S1. One hundred and ninety five genes with the strongest evidence for association with Crohn’s Disease risk, with chromosomal locations, numbers of SNPs, approximate p-values, and Bayes factors**

| **Gene Name** | **Chromosome** | **#SNPs** | **p-value** | **C.BF** |  | **Gene Name** | **Chromosome** | **#SNPs** | **p-value** | **C.BF** |
| --- | --- | --- | --- | --- | --- | --- | --- | --- | --- | --- |
| ISX | 22q12 | 84 | < 3.8×10^-6^ | 148.5 |  | C1orf141** | 1p31 | 10 | < 3.8×10^-6^ | 10.3 |
| SEMA6A* | 5q23 | 152 | < 3.8×10^-6^ | 96.2 |  | EFTUD1 | 15q25 | 16 | < 3.8×10^-6^ | 10.2 |
| GTF3C4 | 9q34 | 4 | < 3.8×10^-6^ | 91.8 |  | MATN1 | 1p35 | 96 | < 3.8×10^-6^ | 10.1 |
| PTGFRN | 1p13 | 15 | < 3.8×10^-6^ | 85.5 |  | LMO4 | 1p22 | 85 | < 3.8×10^-6^ | 9.9 |
| ADRA1B** | 5q33 | 45 | < 3.8×10^-6^ | 82.3 |  | PDIA6 | 2p25 | 12 | < 3.8×10^-6^ | 9.9 |
| MYLK3 | 16q11 | 2 | < 3.8×10^-6^ | 77.0 |  | SOX11 | 2p25 | 194 | < 3.8×10^-6^ | 9.6 |
| HTR3B | 11q23 | 10 | < 3.8×10^-6^ | 75.7 |  | NRG1 | 8p12^+^ | 154 | < 3.8×10^-6^ | 9.2 |
| RRP15 | 1q41 | 29 | < 3.8×10^-6^ | 75.4 |  | GYPA | 4q31 | 22 | < 3.8×10^-6^ | 9.2 |
| RGL1 | 1q25 | 20 | < 3.8×10^-6^ | 69.9 |  | WWC1 | 5q34 | 29 | < 3.8×10^-6^ | 9.1 |
| SORBS1 | 10q23 | 46 | < 3.8×10^-6^ | 65.5 |  | IL12RB2** | 1p31-p31 | 9 | < 3.8×10^-6^ | 9.0 |
| CALCOCO1 | 12q13 | 15 | < 3.8×10^-6^ | 57.9 |  | TRDN | 6q22 | 43 | < 3.8×10^-6^ | 8.9 |
| TMEM156 | 4p14 | 13 | < 3.8×10^-6^ | 52.7 |  | MSGN1 | 2p24 | 9 | < 3.8×10^-6^ | 8.8 |
| XRCC6BP1 | 12q14 | 38 | < 3.8×10^-6^ | 45.9 |  | RBPMS | 8p12^+^ | 16 | < 3.8×10^-6^ | 8.5 |
| FXR1 | 3q28 | 7 | < 3.8×10^-6^ | 37.7 |  | TMEM66 | 8p12^+^ | 28 | < 3.8×10^-6^ | 7.8 |
| GARNL1 | 14q13 | 4 | < 3.8×10^-6^ | 34.9 |  | TTC14 | 3q26 | 9 | < 3.8×10^-6^ | 7.8 |
| GPR161* | 1q24 | 7 | < 3.8×10^-6^ | 30.9 |  | HLA-DQA2* | 6p21 | 17 | < 3.8×10^-6^ | 7.8 |
| SORCS1** | 10q23-q25 | 265 | < 3.8×10^-6^ | 30.6 |  | KATNAL1 | 13q12 | 42 | < 3.8×10^-6^ | 7.7 |
| SAC* | 1q24 | 13 | < 3.8×10^-6^ | 28.4 |  | OTOR | 20p12-p11 | 32 | < 3.8×10^-6^ | 7.7 |
| LRP1B | 2q21 | 241 | < 3.8×10^-6^ | 27.2 |  | VEGFC | 4q34 | 52 | < 3.8×10^-6^ | 7.6 |
| C18orf62 | 18q23 | 79 | < 3.8×10^-6^ | 25.9 |  | FOXI2 | 10q26^+^ | 18 | < 3.8×10^-6^ | 7.6 |
| CSRP1 | 1q32^+^ | 17 | < 3.8×10^-6^ | 24.2 |  | IMPG1 | 6q14-q15 | 48 | < 3.8×10^-6^ | 7.5 |
| POU6F2 | 7p14 | 58 | < 3.8×10^-6^ | 22.6 |  | MAP1B | 5q13 | 36 | < 3.8×10^-6^ | 7.5 |
| LEF1 | 4q23-q25 | 31 | < 3.8×10^-6^ | 22.3 |  | PZP | 12p13-p12 | 29 | < 3.8×10^-6^ | 7.5 |
| SEL1L | 14q31 | 170 | < 3.8×10^-6^ | 21.9 |  | KCTD16 | 5q31 | 51 | < 3.8×10^-6^ | 7.4 |
| SVIP | 11p14^+^ | 88 | < 3.8×10^-6^ | 21.7 |  | COL9A2 | 1p33-p32 | 4 | < 3.8×10^-6^ | 7.4 |
| VRK1 | 14q32 | 128 | < 3.8×10^-6^ | 19.3 |  | PLCL1 | 2q33 | 47 | < 3.8×10^-6^ | 7.1 |
| GLRX3 | 10q26^+^ | 79 | < 3.8×10^-6^ | 18.4 |  | ZNF365** | 10q21 | 54 | < 3.8×10^-6^ | 7.1 |
| ID4* | 6p22 | 79 | < 3.8×10^-6^ | 15.3 |  | KIAA1211 | 4q12 | 32 | < 3.8×10^-6^ | 7.0 |
| CDH10 | 5p14 | 107 | < 3.8×10^-6^ | 14.9 |  | BSN** | 3p21 | 4 | < 3.8×10^-6^ | 7.0 |
| NOD2** | 16q21 | 5 | < 3.8×10^-6^ | 14.6 |  | ZFR** | 5p13 | 19 | < 3.8×10^-6^ | 6.9 |
| NHLRC1* | 6p22 | 7 | < 3.8×10^-6^ | 14.0 |  | LOC100131897 | 5q35 | 26 | < 3.8×10^-6^ | 6.9 |
| FMN2 | 1q43 | 60 | < 3.8×10^-6^ | 14.0 |  | PTER | 10p12 | 39 | < 3.8×10^-6^ | 6.8 |
| IL23R** | 1p31 | 11 | < 3.8×10^-6^ | 13.6 |  | WDFY3 | 4q21 | 17 | < 3.8×10^-6^ | 6.8 |
| PTGER4** | 5p13 | 46 | < 3.8×10^-6^ | 13.5 |  | PAPSS1 | 4q24 | 32 | < 3.8×10^-6^ | 6.7 |
| CTNNA3 | 10q22^+^ | 257 | < 3.8×10^-6^ | 13.3 |  | CRIM1 | 2p21 | 162 | < 3.8×10^-6^ | 6.7 |
| PNPLA6 | 19p13 | 5 | < 3.8×10^-6^ | 13.0 |  | BAMBI | 10p12-p11 | 55 | < 3.8×10^-6^ | 6.6 |
| FBXO15 | 18q22^+^ | 94 | < 3.8×10^-6^ | 12.5 |  | HLCS | 21q22 | 22 | < 3.8×10^-6^ | 6.6 |
| ATG16L1** | 2q37 | 7 | < 3.8×10^-6^ | 12.4 |  | ZNF300** | 5q33 | 7 | < 3.8×10^-6^ | 6.5 |
| RTP2 | 3q27 | 4 | < 3.8×10^-6^ | 12.0 |  | GRM7 | 3p26-p25 | 302 | < 3.8×10^-6^ | 6.5 |
| KCNIP4 | 4p15 | 154 | < 3.8×10^-6^ | 11.8 |  | SIRPD | 20p13 | 7 | < 3.8×10^-6^ | 6.5 |
| SPRED1 | 15q14 | 29 | < 3.8×10^-6^ | 11.2 |  | THUMPD2 | 2p22-p21 | 19 | < 3.8×10^-6^ | 6.5 |
| CYLD** | 16q12 | 30 | < 3.8×10^-6^ | 11.2 |  | NNT | 5p12 | 14 | < 3.8×10^-6^ | 6.4 |
| RAB2A | 8q12 | 10 | < 3.8×10^-6^ | 11.2 |  | DOCK2 | 5q35 | 86 | < 3.8×10^-6^ | 6.4 |
| VCL | 10q22^+^ | 10 | < 3.8×10^-6^ | 11.1 |  | AMT** | 3p21-p21 | 2 | < 3.8×10^-6^ | 6.4 |
| CUX2 | 12q24 | 18 | < 3.8×10^-6^ | 10.9 |  | MCF2L2 | 3q27 | 16 | < 3.8×10^-6^ | 6.4 |
| CADM1 | 11q23 | 124 | < 3.8×10^-6^ | 10.8 |  | FGFR2 | 10q26^+^ | 71 | < 3.8×10^-6^ | 6.3 |
| SIP1 | 14q13 | 2 | < 3.8×10^-6^ | 10.7 |  | SLC22A5* | 5q23 | 4 | < 3.8×10^-6^ | 6.3 |
| SAFB | 19p13-p13 | 3 | < 3.8×10^-6^ | 10.6 |  | JMJD2C | 9p24 | 140 | < 3.8×10^-6^ | 6.3 |
| NAV3 | 12q14 | 122 | < 3.8×10^-6^ | 10.4 |  | LOC441108 | 5q31 | 8 | < 3.8×10^-6^ | 6.3 |

**Table S1 (continued)**

| **Gene Name** | **Chromosome** | **#SNPs** | **p-value** | **C.BF** |  | **Gene Name** | **Chromosome** | **#SNPs** | **p-value** | **C.BF** |
| --- | --- | --- | --- | --- | --- | --- | --- | --- | --- | --- |
| HLA-DRA* | 6p21 | 12 | < 3.8×10^-6^ | 6.3 |  | INO80D | 2q33 | 18 | 1.6×10^-4^ | 4.8 |
| CLSTN2 | 3q23 | 120 | < 3.8×10^-6^ | 6.3 |  | TRPS1 | 8q24 | 98 | 1.7×10^-4^ | 4.8 |
| ACAD11 | 3q22 | 4 | 3.8×10^-6^ | 6.2 |  | DUSP26 | 8p12^+^ | 44 | 1.7×10^-4^ | 4.8 |
| PLOD2 | 3q24 | 78 | 3.8×10^-6^ | 6.2 |  | PTPN2** | 18p11-p11 | 13 | 1.7×10^-4^ | 4.8 |
| NKD1** | 16q12 | 9 | 3.8×10^-6^ | 6.2 |  | FAM65B* | 6p22-p21 | 41 | 2.0×10^-4^ | 4.7 |
| NKX2-3** | 10q24 | 14 | 7.6×10^-6^ | 6.0 |  | GPS2 | 17p13 | 2 | 2.1×10^-4^ | 4.7 |
| GPC5 | 13q32 | 262 | 7.6×10^-6^ | 5.9 |  | RBMS3 | 3p24-p23 | 157 | 2.1×10^-4^ | 4.7 |
| ANKRD27 | 11p15 | 8 | 7.6×10^-6^ | 5.9 |  | RBM47 | 4p14 | 26 | 2.1×10^-4^ | 4.7 |
| MST150** | 5q33 | 5 | 1.5×10^-5^ | 5.8 |  | B3GALTL | 9q22 | 27 | 2.1×10^-4^ | 4.7 |
| NUPL2 | 7p15 | 5 | 1.5×10^-5^ | 5.8 |  | BCL11B | 14q32 | 76 | 2.1×10^-4^ | 4.7 |
| DDX18 | 2q14^+^ | 70 | 3.1×10^-5^ | 5.7 |  | LCOR** | 10q24 | 6 | 2.3×10^-4^ | 4.7 |
| GCC2 | 2q12 | 4 | 3.1×10^-5^ | 5.7 |  | LUZP2 | 11p14^+^ | 161 | 2.3×10^-4^ | 4.7 |
| ZCCHC11 | 1p32 | 7 | 3.1×10^-5^ | 5.7 |  | HCP5* | 6p21 | 19 | 2.3×10^-4^ | 4.7 |
| USH1C | 11p14^+^ | 22 | 3.1×10^-5^ | 5.7 |  | OR11A1* | 6p22-p21 | 8 | 2.6×10^-4^ | 4.6 |
| PCCA | 13q32 | 22 | 3.4×10^-5^ | 5.6 |  | TSHZ1 | 18q22^+^ | 24 | 2.6×10^-4^ | 4.6 |
| DAG1** | 3p21 | 3 | 3.4×10^-5^ | 5.6 |  | SNX7 | 1p21 | 39 | 2.9×10^-4^ | 4.6 |
| IGFBP3 | 7p13-p12 | 70 | 3.4×10^-5^ | 5.6 |  | RPH3A | 15q25 | 23 | 3.1×10^-4^ | 4.5 |
| BANK1 | 4q24 | 34 | 3.4×10^-5^ | 5.6 |  | SLC36A3** | 5q33 | 4 | 3.2×10^-4^ | 4.5 |
| HLA-DQB1* | 6p21 | 7 | 3.4×10^-5^ | 5.6 |  | TUSC3 | 8p22 | 80 | 3.3×10^-4^ | 4.5 |
| SOCS6 | 18q22^+^ | 111 | 3.4×10^-5^ | 5.6 |  | TANC1 | 2q24 | 31 | 3.3×10^-4^ | 4.5 |
| KRTAP2^-4^ | 17q12-q21 | 6 | 3.4×10^-5^ | 5.5 |  | FBXL20 | 17q12 | 4 | 3.5×10^-4^ | 4.5 |
| VAV2 | 9q34 | 27 | 3.8×10^-5^ | 5.5 |  | TFCP2L1 | 2q14^+^ | 30 | 3.5×10^-4^ | 4.5 |
| GIPC2** | 1p31 | 23 | 4.2×10^-5^ | 5.5 |  | CST8 | 20p11 | 9 | 3.6×10^-4^ | 4.5 |
| TMEM86A | 11p15 | 3 | 4.6×10^-5^ | 5.5 |  | CTNNBL1 | 20q11-q12 | 16 | 3.7×10^-4^ | 4.4 |
| TCTA** | 3p21 | 2 | 5.7×10^-5^ | 5.4 |  | PPP2R2C | 4p16 | 31 | 3.7×10^-4^ | 4.4 |
| SLCO6A1 | 5q21 | 15 | 5.7×10^-5^ | 5.4 |  | CERKL | 2q31 | 11 | 3.7×10^-4^ | 4.4 |
| HCG27* | 6p21 | 8 | 6.1×10^-5^ | 5.4 |  | KHDRBS3 | 8q24 | 141 | 3.7×10^-4^ | 4.4 |
| PTCHD2 | 1p36 | 23 | 6.1×10^-5^ | 5.3 |  | EFR3B | 2p23 | 9 | 3.9×10^-4^ | 4.4 |
| PPM1L | 3q26 | 19 | 6.9×10^-5^ | 5.3 |  | C7orf33* | 7q36 | 15 | 4.5×10^-4^ | 4.3 |
| PAPPA | 9q31 | 65 | 7.6×10^-5^ | 5.3 |  | MTTP | 4q24 | 9 | 4.5×10^-4^ | 4.3 |
| ZPLD1 | 3q12 | 76 | 7.6×10^-5^ | 5.3 |  | CCDC93 | 2q14^+^ | 15 | 4.5×10^-4^ | 4.3 |
| TMEM183A | 1q32^+^ | 10 | 7.6×10^-5^ | 5.3 |  | PTRF | 17q21 | 4 | 4.6×10^-4^ | 4.3 |
| LRRC3B | 3p24 | 59 | 7.6×10^-5^ | 5.2 |  | ZAK | 2q24 | 18 | 4.7×10^-4^ | 4.3 |
| C6orf10* | 6p21 | 14 | 7.6×10^-5^ | 5.2 |  | USP4** | 3p21 | 3 | 4.7×10^-4^ | 4.3 |
| MSC | 8q21 | 44 | 7.6×10^-5^ | 5.2 |  | CDK5RAP1 | 20pter-q11 | 6 | 4.7×10^-4^ | 4.3 |
| RNF145** | 5q33 | 9 | 7.6×10^-5^ | 5.2 |  | PTS | 11q22 | 29 | 5.2×10^-4^ | 4.3 |
| GANC | 15q15 | 9 | 8.0×10^-5^ | 5.2 |  | SLC22A4 | 5q31 | 5 | 5.5×10^-4^ | 4.2 |
| YWHAE | 17p13 | 6 | 8.0×10^-5^ | 5.2 |  | TMEM196 | 7p21 | 18 | 5.5×10^-4^ | 4.2 |
| SPAG16 | 2q34 | 74 | 8.0×10^-5^ | 5.1 |  | RNF219 | 13q31 | 34 | 5.6×10^-4^ | 4.2 |
| TIAM1 | 21q22 | 68 | 8.4×10^-5^ | 5.1 |  | ANXA5 | 4q27 | 20 | 5.6×10^-4^ | 4.2 |
| NEK2 | 1q32^+^-q41 | 11 | 8.8×10^-5^ | 5.1 |  | PPM1K | 4q22 | 13 | 5.6×10^-4^ | 4.2 |
| GRM8 | 7q31-q32 | 112 | 9.2×10^-5^ | 5.1 |  | ZNF680 | 7q11 | 54 | 5.7×10^-4^ | 4.2 |
| FOSL2 | 2p23 | 9 | 9.5×10^-5^ | 5.0 |  | FCRL4 | 1q21 | 7 | 5.9×10^-4^ | 4.2 |
| ADO** | 10q21 | 8 | 9.5×10^-5^ | 5.0 |  | CEBPB | 20q13 | 15 | 5.9×10^-4^ | 4.2 |
| ERBB4 | 2q33-q34 | 153 | 1.1×10^-4^ | 5.0 |  | C10orf57 | 10q22^+^ | 6 | 6.3×10^-4^ | 4.2 |
| MGA | 15q14 | 5 | 1.1×10^-4^ | 5.0 |  | SGK1* | 6q23 | 46 | 6.3×10^-4^ | 4.1 |
| ZNF717 | 3p12 | 41 | 1.3×10^-4^ | 4.9 |  | NRXN1 | 2p16.3 | 221 | 6.4×10^-4^ | 4.1 |
| KIAA0146 | 8q11 | 21 | 1.6×10^-4^ | 4.8 |  | SYT7 | 11q12-q13 | 8 | 6.4×10^-4^ | 4.1 |
| ZNF25 | 10p11 | 7 | 1.6×10^-4^ | 4.8 |  |  |  |  |  |  |

** indicates genes in the chromosomal locations where the WTCCC single-SNP analysis showed **strong** evidence.

* indicates genes in the chromosomal locations where the WTCCC single-SNP analysis showed **moderate** evidence.

+ indicates chromosomal locations are those with three or more genes in the 195 genes showing strong evidence in our WTCCC logic-regression-based analysis, but without strong or moderate evidence in the single-SNP analysis of WTCCC.
