# Supplementary figures and images for: SNP-SNP Interactions Discovered by Logic Regression Explain Crohn's Disease Genetics
Source: PLoS One. 2012 Oct 12;7(10):e43035. doi: 10.1371/journal.pone.0043035 (PMC3470545; doi:10.1371/journal.pone.0043035)

**Cases**

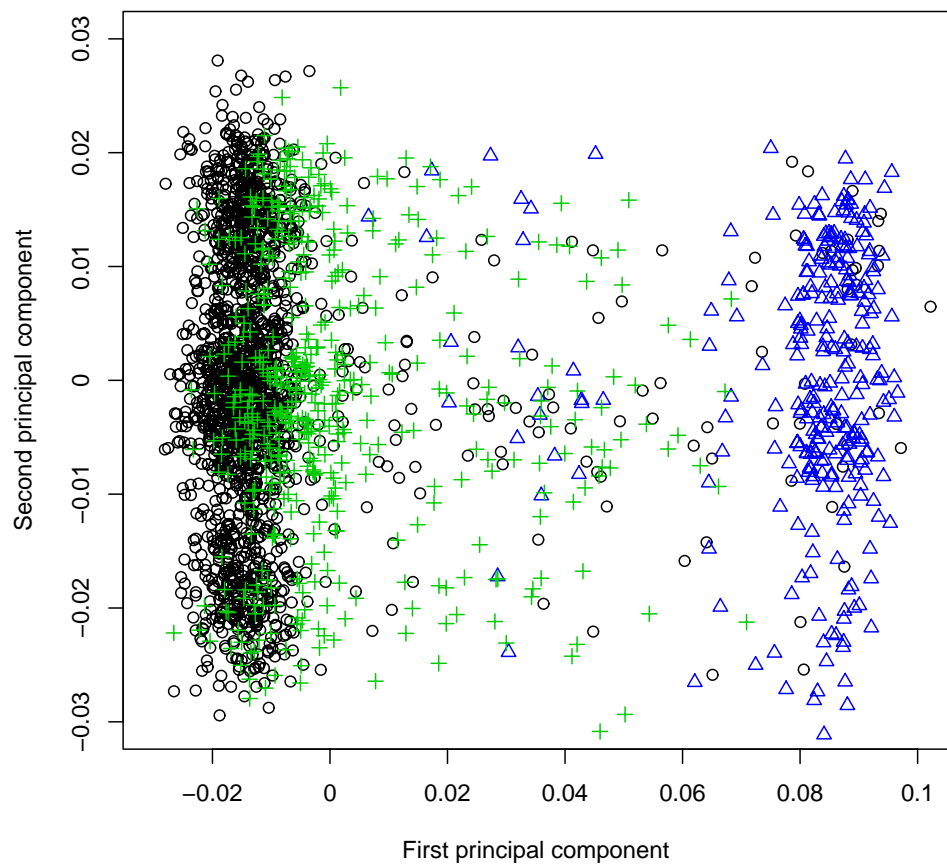

**Controls**

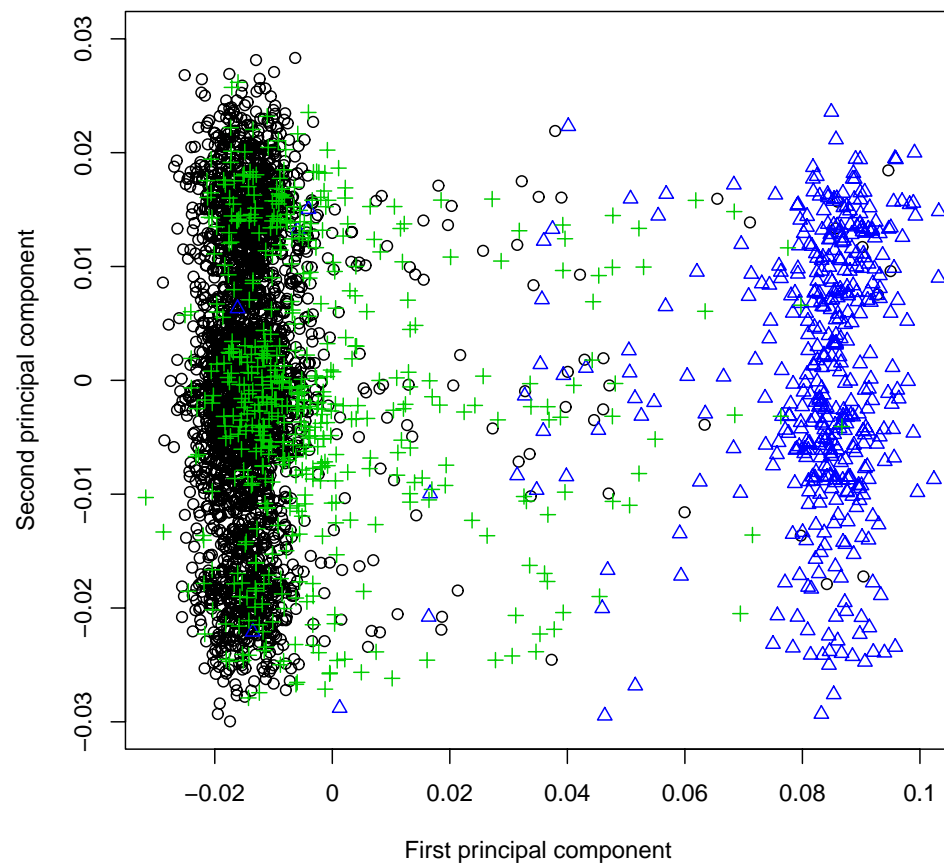

Supplement: Figure S1 — Principal components from the three datasets: WTCCC, Non-Jewish and Jewish subjects are represented by black circles, green pluses, and blue triangles, respectively. (PDF) [file pone.0043035.s002.pdf]
